# Using event-related brain potentials to explore the temporal dynamics of decision-making related to information security

**DOI:** 10.3389/fnins.2022.878248

**Published:** 2022-08-10

**Authors:** Robert West, Bridget Kirby, Kaitlyn Malley

**Affiliations:** ^1^Department of Psychology and Neuroscience, DePauw University, Greencastle, IN, United States; ^2^School of Behavioral and Brain Sciences, University of Texas at Dallas, Richardson, TX, United States

**Keywords:** information security, ethical decision making, event—related potentials (ERP), delay of reward, perspective taking

## Abstract

Insider threat from individuals operating within an organization presents a significant source of violations of information security. Our previous research has used scalp recorded event-related brain potentials (ERPs) and the Information Security Paradigm (ISP) to identify the neural correlates of decision-making processes related to violations of information security. In the current study, we sought to expand this research by examining the effects of two variables that were drawn from the broader decision-making literature (i.e., the benefactor and delay of a reward) on ERPs measured in the ISP. In the ISP we varied whether Josh—a hypothetical IT specialist—or a significant other was the benefactor of a violation, and whether the benefit of a violation was received after a short or long delay. The choice data revealed that individuals were less likely to endorse an unethical action than a control action. The electrophysiological data revealed ERPs that differentiated ethical scenarios from control scenarios between 200 and 2,000 ms after onset of the decision prompt, distributed over the occipital, central, and lateral frontal regions of the scalp. These ERPs were insensitive to the benefactor and delay of the reward. In contrast, there was slow wave activity over the frontal-polar region that was sensitive to both variables. The current findings provide evidence for separable neural systems that are either generally related to ethical decision-making in the ISP or are sensitive to the benefactor or delay of a reward resulting from an unethical decision.

## Introduction

In the context of information security, insider threat represents intentional or accidental violations of information security policies that result from the action(s) of a person or persons operating inside of an organization (Balozian and Leidner, 2017; Bailey et al., 2018). Managers of information technology resources recognize insider threat as a significant security concern that is second only to major computer viruses (Ernst and Young, 2010), and instances of insider threat may contribute to 50% of violations of information security (Richardson, 2011). The significance of insider threat has led information system researchers to explore system variables (e.g., the severity of punishment) and personal traits (e.g., self-control or moral belief) that predict violations of information security (Zhang et al., 2009; Vance and Siponen, 2012; Balozian and Leidner, 2017; Luo et al., 2020). Our own research has used event-related brain potentials (ERPs) to explore the neural correlates of ethical decision-making related to insider threat (Hu et al., 2015; West et al., 2019). The current project builds upon this work, by examining the effects of two variables (i.e., the benefactor and delay of a reward) on ERP activity in the Information Security Paradigm (ISP). These variables were drawn from the moral and economic decision-making literatures, as both are important determinants of decision-making and consistently associated with neural recruitment in the anterior frontal regions (D’Argembeau et al., 2007; Mitchell et al., 2010; Kable, 2014).

The ISP (Hu et al., 2015) was developed to explore the neural correlates of decision-making related to information security using ERPs. The measure is founded in more traditional work in information security (Hu et al., 2011), and has also been adapted for use with fMRI (Duan et al., 2021). In the task, individuals are presented with various scenarios and prompts and asked to rate how likely they would be to engage in a given behavior. Control items represent decisions that do not involve an ethical element (e.g., assisting a colleague with a project), while violation items represent decisions that involve an ethical violation related to information security (e.g., unauthorized access of a secure server). Individuals are more likely to endorse (respond Yes) to control items than violation items demonstrating a general preference for avoiding unethical behavior, with this preference being moderated by the severity of the violation (Hu et al., 2015; West et al., 2019).

Studies using ERPs with the ISP reveal that decision-making in the task is associated with modulations of the physiology that differentiate ethical violation items from control items between 200 and 2,000 ms after onset of the decision prompt (Hu et al., 2015; West et al., 2019; West and Cowger, 2021). The earliest effect of an ethical violation on the ERPs reflects a reduction in the amplitude of the posterior N2 component that may result from an inward focus of attention for violation items that limits visual processing of the prompt (West and Cowger, 2021). Between 400 and 2,000 ms the ERPs differentiate ethical items from control items over the lateral frontal, medial central, and lateral central regions of the scalp (Hu et al., 2015; West et al., 2019). These findings indicate that ethical decision making in the ISP is associated with the recruitment of a temporally and spatially distributed neural network, consistent with broader literature on the neural foundation of moral decision-making (Greene et al., 2001; Young et al., 2007; Greene and Young, 2020).

Hu et al. (2015) demonstrated that some of these modulations of the ERPs were sensitive to both the presence and severity of the ethical violation, although the effect of severity was not replicated in a later study (West et al., 2019). The effect of an ethical violation on the longer latency modulations of the ERPs is sensitive to individual differences in self-control and moral beliefs (Hu et al., 2015; West et al., 2019). Consistent with the dual processes account of moral decision-making (Greene et al., 2001, 2008), slow wave activity in the ISP is greater in those with high self-control (Hu et al., 2015; West et al., 2019) and reduced in those with high moral belief (West et al., 2019). These findings converge with behavioral evidence demonstrating that both traits are reliable predictors of decision-making related to information security (Myyry et al., 2009; Siponen et al., 2012; Li et al., 2018).

One issue that remains unclear is whether the ERPs observed in the ISP reflect activity within neural systems that are generally related to ethical decision-making or that contribute to the processing of specific attributes of the ethical dilemma. Given this, the current study had two goals: First, we sought to provide a conceptual replication of our prior research using a new set of materials in the ISP. Second, we sought to broaden our understanding of the neuro-cognitive processes contributing to ethical decision-making in this domain by examining the influence of two independent variables (i.e., the benefactor and delay of a reward) drawn from the broader literature on moral and economic decision making that are relevant in the context of information security (Balozian and Leidner, 2017). We expected the ERPs over the occipital, central, and lateral frontal regions of the scalp to distinguish ethical items from control items. The literature examining the neural foundation of self-referent processing (D’Argembeau et al., 2007; Decety and Porges, 2011) and intertemporal choice (Mitchell et al., 2010) lead to the prediction that the ERPs over the frontal region will be sensitive to the benefactor and delay of a reward.

## Methods and materials

### Transparency and openness

The behavioral and physiological data used in the analyses are available at osf.io/f9dbv along with the materials for the ISP. Data for forty of the participants reported in this article were included in Kirby et al. (2019). The current work provides an expanded sample and more complete analysis of this dataset.

### Participants

Seventy individuals enrolled in introductory psychology courses completed the study. The EEG data for two individuals had a high degree of artifact and could not be used, and the behavioral data for one additional participant was lost due to a computer error. Sixty-seven individuals (*M* = 19.88, *SD* = 2.80 years of age; Female = 34, Male = 33; White = 47, Asian = 8, Hispanic or Latinx = 6, Black = 2, Indigenous = 2, Other = 2) provided complete and usable data for the study. On average participants had completed *M* = 1.42, *SD* = 1.63 years of university coursework.

### Information security paradigm

**FIGURE 1:**
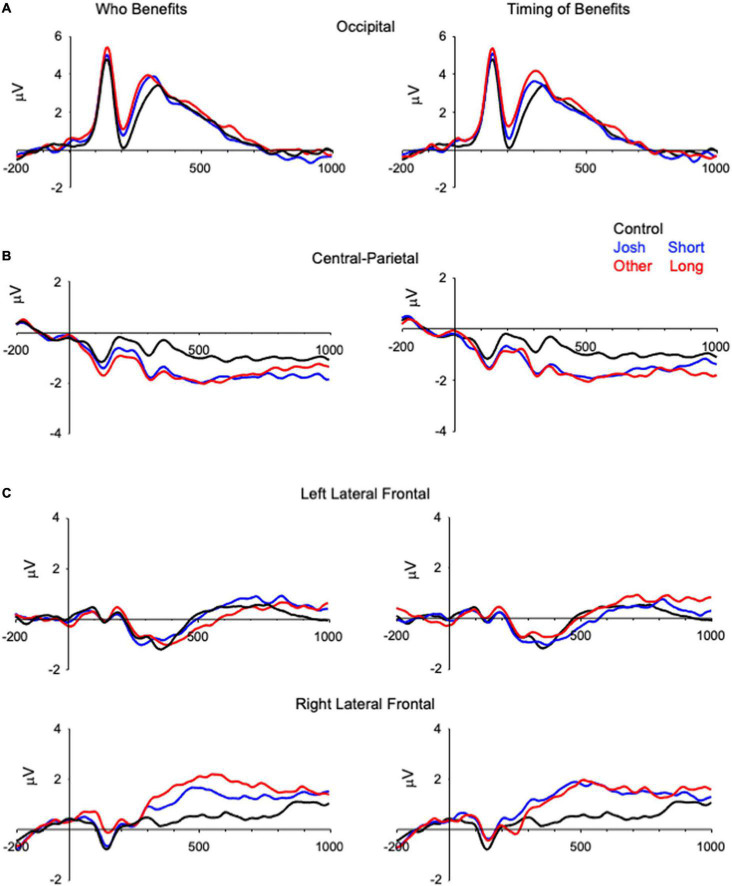
Grand-averaged ERPs demonstrating differences in the physiology between the two types of trials (i.e., ethical vs. control) that are averaged across the electrodes included in the analyses (e.g., for the N2 the plotted electrode is the average of O1, Oz, O2). The left side of the figure plots Control, Josh, and Other items, and the right side of the figure plots Control, Short and Long delay items. **(A)** N2, **(B)** central-parietal negativity, and **(C)** right frontal-temporal positivity from −200 ms before onset of the prompt to 1,000 ms after onset of the prompt. The y-axis represents onset of the prompt and the minor x-axis tick marks represent 100 ms increments.

In the ISP individuals were instructed to respond as if they were Josh, an IT specialist with extensive knowledge of the company’s information systems. The ISP reflected a 3 (Benefactor: Control, Josh, Other) × 2 (Delay of reward: Short, Long) factorial with eight scenarios for each of the six cells of the design. The full set of materials is available online at the open science portal. Control scenarios were items involving a decision that was unrelated to an ethical violation. Josh benefit scenarios involved behaviors wherein he was rewarded for the unethical choice, and Other benefit scenarios involved behaviors wherein a friend, relative or partner was rewarded for his unethical choice. For the Other benefit scenarios, the name and relationship of the Other person was stated in the scenario. In Short delay scenarios, the reward was received after 0–3 months; and for Long delay scenarios, the reward was received after 12–14 months. The 48 scenarios and prompts were presented in a different random order for each individual that was generated by the PsychoPy software. Scenarios were limited to 300 characters and prompts were 22–50 characters. Prompts were posed in the form of a question. The prompts did not mention the benefactor or delay of the reward. Individuals were given an unlimited amount of time to read the scenario and then pressed the spacebar to view the prompt. A fixation (+) was presented for 500 ms between the scenario and prompt. The response time and EEG data were time locked to the onset of the prompt (c.f., Figure 1; West et al., 2019). Individuals responded with a four-point scale (No, Likely No, Likely Yes, Yes) using the C-V-B-N keys on the keyboard. Participants were instructed to rest their fingers on the keys during the trials to reduce movement artifacts. The scenarios and prompts were presented in white font on a black ground and were centered on the screen with the text being left justified.

### Procedure

Upon arriving at the laboratory individuals were given a brief overview of the procedure and signed informed consent was obtained. Individuals then completed a demographic survey and several questionnaires measuring individual differences related to self-control, moral foundations, media exposure, pathological gaming, and grit. Following completion of the scales, individuals were fitted with a 32 electrode actiCAP and completed the ISP, a modified version of the moral foundations task, and a picture rating task while EEG was recorded. Then the EEG cap was removed, individuals were debriefed, and compensated with either course credit or $15.

### EEG recording and analysis

The EEG was recorded at 500 Hz with a 32 channel actiCHamp system using the Brain Vision Recorder Software and a standard actiCAP with electrodes CP5-CP6 replaced with active electrodes located below the eyes. The electrodes were grounded to Fpz and referenced to Cz during recording. The EEG data were bandpass filtered between 0.1 and 30 Hz using an IIR filter (ERPLAB 5.1.1.0; Lopez-Calderon and Luck, 2014) and referenced to an average reference following the correction of ocular artifacts (blinks and saccades) using ICA EEGLAB (13.6.5b) (Delorme and Makeig, 2004). Trials including other artifacts were rejected before averaging using a ± 100 μV threshold. ERPs were averaged for Control, Josh benefit, Other benefit, Short delay, or Long delay items from −200 to 2,000 ms around onset of the prompt. Measures of mean voltage were made using the measurement tool in ERPLAB.

The effects of Benefactor and Delay of Reward on the ERPs were examined in a set of 2 or 3 factor ANOVAs comparing the ERPs for Control, Josh or Other benefit items, or Control, Short or Long delay items, while collapsing across the other independent variable. Hemisphere was included as a factor in analyses when lateralized ERPs were expected based upon previous research, and electrodes and measurement epochs included in the analyses were based upon previous research with the ISP (Hu et al., 2015; West et al., 2019; West and Cowger, 2021). Region was included in one of the exploratory analyses based upon the pattern observed in the ERP data. The *a priori* analyses included: N2 (O1-Oz-O2; 200–300 ms), left frontal temporal (T8-F8-FT10/T7-F7-FT9 400–800 ms), central-parietal negativity (Cz-Pz-CP1-CP2, 300–600 ms), central-slow wave (FC2-C4-CP2/C3-C3-CP1, 400–1,800 ms). The exploratory analyses included: anterior frontal region for Benefactor (Fp1-Fp2, F3-F4, 800–1,600 ms), and anterior frontal for Delay (Fp1-Fp2, FT9-FT10). We report inferential statistics for the main effect of condition and the condition by hemisphere/region interaction where relevant; the main effect of electrode is not reported as this effect simply captures differences in amplitude across electrodes that are independent of condition.

## Results

### Psychometric data

To establish the reliability of the ISP we estimated Cronbach’s α for the Control, Josh and Other benefit items. Reliability for Control items was modest (α = 0.63, best = 0.64) and dropping any of the individual items did not result in improvement. For Josh benefit (α = 0.91, best = 0.91) and Other benefit (α = 0.88, best = 0.90) items the reliability was high, with one item (Other short 2) being negatively related to the remaining Other benefit items. These data indicate that the ISP demonstrates acceptable internal consistency, and that future work could focus on refining a subset of the items.

### Behavioral data

The behavioral data are presented in Table 1. Choice for the Josh items was significantly correlated with choice for the Other items (*r* = 0.80, *p* < 0.001). In contrast, choice for the Control items was not significantly correlated with either the Josh (*r* = 0.07, *p* = 0.57) or Other (*r* = 0.18, *p* = 0.14) items. These findings are consistent with West et al. (2019) in demonstrating that ethical violation items and control items may reflect different constructs.

**TABLE 1:** Mean and standard deviation for choice and response time (milliseconds) for the ISP.

		Control	Josh	Other
Choice	Short	2.93 (0.41)	1.65 (0.52)	1.72 (0.46)
	Long	2.94 (0.29)	1.95 (0.61)	1.65 (0.54)
Response time	Short	1,877 (797)	1,676 (831)	1,853 (847)
	Long	1,963 (683)	2,054 (955)	1,759 (810)

A pair of 3 (Benefactor: Control, Josh, Other) × 2 (Delay: Short or Long) ANOVAs was used to examine the behavioral data. For the choice data, the main effect of Benefactor was significant, *F*(2, 132) = 269.35, *p* < 0.001, η^2^*_*G*_* = 0.58, with individuals being more likely to say yes for Control items than Josh or Other items; the main effect of Delay was significant, *F*(1, 66) = 7.97, *p* = 0.006, η^2^*_*G*_* = 0.006, with individuals being more likely to say yes for the Long delay items; and the main effects were qualified by an interaction, *F*(2, 132) = 20.49, *p* < 0.001, η^2^_*G*_ = 0.027. A simple main effects test revealed that the effect of Delay was significant for Josh, *F*(1, 66) = 43.94, *p* < 0.001, and possibly Other, *F*(1, 66) = 3.99, *p* = 0.05, items, but not for Control items, *F* < 1.00.

For the response time data, the main effect of Benefactor was not significant, *F* < 1.00; the main effect of Delay was significant, *F*(1, 66) = 5.59, *p* = 0.021, η^2^*_*G*_* = 0.006, with individuals taking longer to respond for the Long delay items; and the interaction was significant, *F*(2, 132) = 6.73, *p* = 0.002, η^2^*_*G*_* = 0.014. A simple main effect test revealed that response time was slower for Long delay than Short delay items when Josh was the benefactor, while the effect of Delay was not significant for the Other, *F* < 1.00, or Control, *F*(1, 66) = 1.02, *p* = 0.32, items.

### Event-related brain potentials data

**FIGURE 2:**
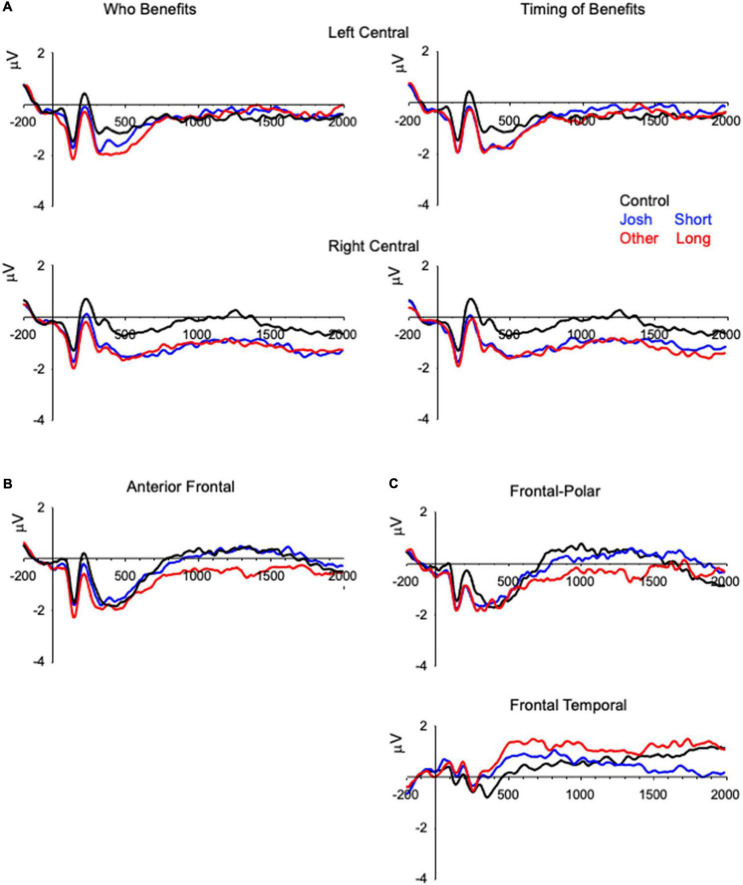
Grand-averaged ERPs demonstrating differences in the physiology between the two types of trials (i.e., ethical vs. control) that are averaged across the electrodes included in the analyses. **(A)** The right central slow wave differentiating control items from ethical violation items, **(B)** anterior frontal slow wave differentiating other items from Josh and control items, and **(C)** the frontal-polar/frontal temporal slow wave differentiating long delay items from control and short delay items. The ERPs are plotted from 200 ms before onset of the prompt to 2,000 ms after onset of the prompt, and the y-axis represents onset of the prompt.

A subset of the ERP data is presented in Figures 1, 2. Consistent with previous research (Hu et al., 2015; West et al., 2019), these data reveal modulations of the ERPs that distinguish ethical from control items between 200 and 2,000 ms after onset of the prompt. These data also reveal slow wave activity over the anterior frontal region that was sensitive to the benefactor and delay of the reward (Figures 2B,C).

#### N2

The analysis of Benefactor revealed a significant effect of Condition, *F*(2, 132) = 11.26, *p* < 0.001, η^2^*_*G*_*, with Control items demonstrating greater negativity than Josh (*t* = 3.29, *p* = 0.004) or Other (*t* = 4.61, *p* < 0.001) items (Figure 1A and Table 2), that did not differ from one another (*t* = 1.32, *p* = 0.57). The main effect of Condition was also significant in the analysis of Delay, *F*(2, 132) = 11.01, *p* < 0.001, η^2^*_*G*_* = 0.031, with Control items revealing greater negativity than Short (*t* = 2.93, *p* = 0.012) and Long (*t* = 4.64, *p* < 0.001) delay items, that did not differ from one another (*t* = 1.70, *p* = 0.27).

**TABLE 2:** Mean and standard deviation of voltage in microvolts for effects of Benefactor and Delay of Reward for modulations of the ERPs that were sensitive to ethical decision-making.

		Control	Josh	Other	Short	Long
N2		1.29 (2.78)	2.20 (2.96)	2.57 (2.78)	2.14 (2.81)	2.64 (3.04)
RFTP	Left	0.26 (2.52)	0.35 (2.78)	−0.04 (2.78)	0.02 (2.82)	0.33 (2.85)
	Right	0.47 (2.19)	1.37 (2.54)	1.88 (3.06)	1.61 (2.46)	1.63 (2.80)
CPN		−0.82 (1.92)	−1.77 (1.90)	−1.88 (2.07)	−1.78 (1.84)	−1.86 (2.08)
CSW	Left	−0.64 (1.57)	−0.57 (1.60)	−0.67 (1.71)	−0.55 (1.54)	−0.70 (1.68)
	Right	−0.33 (1.41)	−1.22 (1.52)	−1.20 (1.73)	−1.16 (1.61)	−1.26 (1.57)
Delay of	FP	0.42 (3.51)			0.28 (2.63)	−0.38 (2.93)
Benefit	FT	0.65 (2.30)			0.54 (2.39)	1.13 (2.99)

Left and Right represent the left and right hemispheres; FP represents the frontal-polar region and FT represents the frontal temporal region.

#### Central-parietal negativity (CPN)

In the Benefactor analysis the effect of Condition was significant, *F*(2, 132) = 16.90, *p* < 0.001, η^2^_*G*_ = 0.042; Josh (*t* = 4.74, *p* < 0.001) and Other (*t* = 5.29, *p* < 0.001) items revealed greater negativity than Control items (Figure 1B and Table 2), and did not differ from one another (*t* = 0.55, *p* = 1.00). The analysis of Delay revealed a main effect of Condition, *F*(2, 132) = 19.81, *p* < 0.001, η^2^*_*G*_* = 0.043; with Short (*t* = 5.24, *p* < 0.001) and Long (*t* = 0.55, *p* = 1.00) delay items revealing greater negativity than Control items, and did not differ from one another (*t* = 0.40, *p* = 1.00).

#### Right frontal-temporal positivity (RFTP)

In the analysis of Benefactor the effect of Condition was significant, *F*(2, 132) = 6.41, *p* = 0.002, η^2^*_*G*_* = 0.005, and was qualified by a Condition × Hemisphere interaction, *F*(2, 132) = 6.00, *p* = 0.0003, η^2^*_*G*_* = 0.011, (Figure 1C); the effect of Condition was significant for the right, *F*(2, 132) = 10.23, *p* < 0.001, but not left, *F*(2, 132) = 1.06, *p* = 0.35, hemisphere. For the right hemisphere, Control items differed from Josh (*t* = 2.84, *p* = 0.016) and Other (*t* = 4.47, *p* < 0.001) items, that did not differ from one another (*t* = 1.63, *p* = 0.32). The analysis for Delay revealed an effect of Condition, *F*(2, 132) = 9.088, *p* < 0.001, η^2^*_*G*_* = 0.006, and a Condition × Hemisphere interaction, *F*(2, 132) = 4.56, *p* = 0.012, η^2^*_*G*_* = 0.008, right hemisphere, *F*(2, 132) = 12.35, *p* < 0.001), left hemisphere, *F* < 1.00. For the right hemisphere, Control items differed from Short (*t* = 4.27, *p* < 0.001) and Long (*t* = 4.34, *p* < 0.001) delay items, that did not differ from one another (*t* = 0.08, *p* = 1.00).

#### Central slow wave (CSW)

The analysis of Benefactor revealed an effect of Condition, *F*(2, 132) = 5.53, *p* = 0.005, η^2^*_*G*_* = 0.01, and a Condition × Hemisphere interaction, *F*(2, 132) = 12.35, *p* < 0.001, η^2^*_*G*_* = 0.011 (Figure 2A). A simple main effect test revealed that the effect was significant for the right, *F*(2, 132) = 13.95, *p* < 0.001, but not left, *F* < 1.00, hemisphere. For the right hemisphere, the ERPs for Control items were less negative than those for Josh (*t* = 4.61, *p* < 0.001) or Other (*t* = 4.53, *p* < 0.001) items, that did not differ from one another (*t* = 0.08, *p* = 1.00). The analysis of Delay revealed a main effect of Condition, *F*(2, 132) = 6.32, *p* = 0.002, η^2^*_*G*_* = 0.011, and a Condition × Hemisphere interaction, *F*(2, 132) = 16.79, *p* < 0.001, η^2^*_*G*_* = 0.011; and the effect of condition was significant for the right, *F*(2, 132) = 16.11, *p* < 0.001, but not left, *F* < 1.00, hemisphere. For the right hemisphere, the ERPs for Control items were less negative than those for Short (*t* = 4.62, *p* < 0.001) or Long (*t* = 5.17, *p* < 0.001) delay items, that did not differ from one another (*t* = 0.56, *p* = 1.00).

#### Benefactor of reward

The analysis of the anterior frontal slow wave activity that was sensitive to benefactor revealed a significant main effect of Condition, *F*(2, 132) = 5.28, *p* = 0.006, η^2^*_*G*_* = 0.013 (Figure 2B), that reflected greater negativity for Other items (*M* = −0.50 μV) than for Josh items (*M* = 0.20 μV, *t* = 2.60, *p* = 0.031) or for Control items (*M* = 0.30 μV, *t* = 2.99, *p* = 0.01), that did not differ from one another (*t* = 0.39, *p* = 1.00).

#### Delay of reward

The analysis of slow wave activity that was sensitive to timing of the benefit revealed a Condition × Region interaction, *F*(2, 132) = 3.94, *p* = 0.022, η^2^*_*G*_* = 0.007 (Figure 2C and Table 2). Follow-up analyses revealed that Short delay and Control items did not differ significantly from one another, Condition × Region *F* < 1.00, while Long delay items differed from Short delay, *F*(2, 132) = 6.38, *p* = 0.014, η^2^*_*G*_* = 0.008, and Control, *F*(2, 132) = 5.25, *p* = 0.025, η^2^*_*G*_* = 0.008, items.

## Discussion

The behavioral data reveal some noteworthy findings. The ISP demonstrated good reliability that was stronger for the violation items than the control items, providing a novel contribution to the literature. Choice behavior was highly correlated for Josh and Other items, that were in turn weakly correlated with Control items. Together, the reliability and correlations reveal that the ISP provides a robust measure of ethical decision-making that can be distinguished from more general decision-making realized in the Control items. The choice data revealed that individuals were less likely to respond yes to ethical items than control items, extending prior findings (Hu et al., 2015; West et al., 2019), and providing a measure of confidence that individuals are engaged in the task, as random responding might be expected to result in similar choice behavior across the ethical and control items.

The ERP data revealed four patterns relevant to the goals of the study that were to provide a conceptual replication of the ISP with a new set of materials, and to examine the possible effects of the benefactor and delay of a reward in the ISP. The amplitude of the posterior N2, right frontal temporal positivity, and central parietal negativity differed for violation items and control items, and the nature of the difference was similar to that observed in research using the original ISP materials (Hu et al., 2015; West et al., 2019). Together, these findings may indicate that the three modulations of the ERP reveal activity in neural systems that are generally related to ethical decision-making in the ISP, being rather insensitive to the characteristics of the ethical dilemmas.

The ERP data also revealed slow wave activity over the right central region between 400 and 1,800 ms that distinguished ethical and control items. This finding can be contrasted with the findings of West et al. (2019) who observed slow wave activity over the left central region in a similar time period. The reason for this difference is unclear. The studies were conducted using the same equipment and processing pipeline, so this seems unlikely to be driving the difference, while the materials did differ across studies. A second study using the Josh and Control items from the current task also revealed slow wave activity over the right central region for ethical than control items (West and Cowger, 2021). Given this, there may be some value in comparing the two sets of materials in the same individuals in an effort to distinguish robust findings from idiosyncratic characteristics of a dataset or set of materials.

We also observed slow wave activity over the anterior frontal region that was sensitive to the benefactor and delay of reward. The slow wave activity that was sensitive to benefactor distinguished Other items from Josh and Control items. This difference may indicate that the slow wave activity represents the engagement of a neuro-cognitive process supporting perspective taking that is recruited when contemplating an unethical action that benefits another individual rather than the self (D’Argembeau et al., 2007). The slow wave activity that was sensitive to the delay of the benefit distinguished Long delay items from Short delay and Control items. The anterior frontal distribution of the ERP activity would be consistent with the finding that the orbital and ventromedial prefrontal cortex is recruited during decision-making involving the consideration of delayed gains in the context of temporal discounting (Mitchell et al., 2010). Generally, these findings converge with ideas from the moral reasoning literature wherein moral decision-making is thought to emerge from the recruitment of general-purpose neuro-cognitive-affective mechanisms that are brought to bear on the problem at hand rather than specialized neural systems that are unique to moral reasoning (Greene and Young, 2020).

There are some limitations of the current study that should be considered. First, the sample primarily included undergraduates in the first 2 years of study that likely had limited experience with information security policies, so it could be interesting to explore neural activity in the ISP in individuals with greater knowledge of and experience with information security policy. Second, the study did not consider the impact of individual difference variables (e.g., self-control or moral belief) that are known to influence neural activity (Hu et al., 2015; West et al., 2019) and decision-making related to information security (Zhang et al., 2009; Siponen et al., 2012). The convergence between the current and other published findings have begun to reveal reliable modulations of the ERPs related to decision-making in the ISP that could serve as the foundation to address these limitations in future studies.

## Conclusion

In conclusion, the findings of the current study demonstrate that ethical decision-making related to information security as measured in the ISP represents a reliable construct that can be distinguished from more general decision-making contributing to Control trials. The ERP data reveal that ethical decision-making in the ISP is associated with the recruitment of neuro-cognitive processes that operate in parallel beginning shortly after onset of the decision prompt and ending around the time of the behavioral response; with some evidence indicating that the right hemisphere may be preferentially engaged in decision-making within the task. The findings also reveal that ethical decision-making may be supported by the recruitment of processes that are either domain specific (i.e., distinguishing ethical from control trials) or more generally related to information processing that is relevant to the decision context (i.e., effects of benefactor and delay of the reward).

## Data availability statement

The datasets presented in this study can be found in online repositories. The names of the repository/repositories and accession number(s) can be found below: Open Science Framework—osf.io/f9dbv.

## Ethics statement

The studies involving human participants were reviewed and approved by the Institutional Review Board, DePauw University. The patients/participants provided their written informed consent to participate in this study.

## Author contributions

RW, BK, and KM contributed to study design, data collection and analysis, and manuscript preparation. All authors contributed to the article and approved the submitted version.
